# Data on dopant characteristics and band alignment of CdTe cells with and without a ZnO highly-resistive-transparent buffer layer

**DOI:** 10.1016/j.dib.2018.12.002

**Published:** 2018-12-06

**Authors:** G. Kartopu, B.L. Williams, V. Zardetto, A.K. Gürlek, A.J. Clayton, S. Jones, W.M.M. Kessels, M. Creatore, S.J.C. Irvine

**Affiliations:** a Centre for Solar Energy Research, OpTIC, Swansea University, St. Asaph Business Park, LL17 0JD, UK; b Department of Applied Physics, Eindhoven University of Technology, 5600 MB, The Netherlands

## Abstract

Photovoltaic enhancement of cadmium telluride (CdTe) thin film solar cells using a 50 nm thick, atomic-layer-deposited zinc oxide (ZnO) buffer film was reported in “Enhancement of the photocurrent and efficiency of CdTe solar cells suppressing the front contact reflection using a highly-resistive ZnO buffer layer” (Kartopu et al., 2019) [1].

Data presented here are the dopant profiles of two solar cells prepared side-by-side, one with and one without the ZnO highly resistive transparent (HRT) buffer, which displayed an open-circuit potential (V_oc_) difference of 25 mV (in favor of the no-buffer device), as well as their simulated device data. The concentration of absorber dopant atoms (arsenic) was measured using the secondary ion mass spectroscopy (SIMS) method, while the density of active dopants was calculated from the capacitance-voltage (CV) measurements. The solar cell simulation data was obtained using the SCAPS software, a one-dimensional solar cell simulation programme. The presented data indicates a small loss (around 20 mV) of V_oc_ for the HRT buffered cells.

**Specifications table**

**Table:** 

Subject area	Physics
More specific subject area	Photovoltaic Solar Cells
Type of data	Figure
How data was acquired	SIMS (Cameca IMS-4f), C-V (Solartron impedance analyzer), SCAPS simulation tool
Data format	Raw, analyzed
Experimental factors	Before SIMS diluted bromine chemical etch (surface-polish); before C-V measurements gold back contact deposition on sample surface
Experimental features	SIMS indicated arsenic dopant concentration and distribution and analysis of C-V data provided net carrier density in the absorber film; SCAPS simulated the band-alignment and cell performance
Data source location	LSA Ltd., Loughborough, UK (SIMS); Center for Solar Energy Research, OpTIC, Swansea University, St Asaph, UK (C-V, SCAPS)
Data accessibility	All data are presented in this article
Related research article	G. Kartopu, B.L. Williams, V. Zardetto, A.K. Gürlek, A.J. Clayton, S. Jones, W.M.M. Kessels, M. Creatore, S.J.C. Irvine, Enhancement of the photocurrent and efficiency of CdTe solar cells suppressing the front contact reflection using a highly-resistive ZnO buffer layer, Solar Energy Materials and Solar Cells, 191 (2019) 78–82 [1]

**Value of the data**

• The SIMS can provide arsenic density in CdTe:As at a detection limit of ~1 × 10^16^ As/cm^3^
• The C-V curves (1/C^2^ vs. V) can be analyzed to estimate the acceptor density in the absorber
• Ratio of the acceptor density (C-V result) to dopant atom density (SIMS result) provides an estimate of efficiency of dopant activation
• The good sensitivity of SIMS and C-V methods makes them powerful in investigating doping-related issues with CdTe thin film solar cells
• SCAPS helps to visualize the band-alignment, and to quickly assess influence of various material parameters on the cell performance, guiding experimental solar cell research.

## 1 Data

**Fig. 1:**
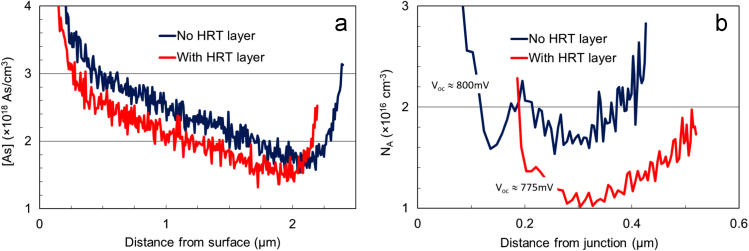
(a) SIMS arsenic concentration [As] and (b) CV-derived acceptor density (*N*_A_) for two solar cell structures, with and without the ZnO HRT layer. Less As is incorporated, and the corresponding *N*_A_ is lower for the CdTe cell with HRT buffer. Measured device *V*_oc_׳s are indicated in (b).

Fig. 1a shows the distribution of As dopant atoms within the solar cells’ CdTe absorber layer. It shows that less As is incorporated for the sample containing the ZnO HRT buffer film. The net acceptor density (*N*_A_), shown in Fig. 1b, tracks the As profiles in Fig. 1a in that the *N*_A_ for the cell with HRT buffer is lower (~1.0 × 10^16^ vs. 1.7 × 10^16^ cm^-3^).

**Fig. 2:**
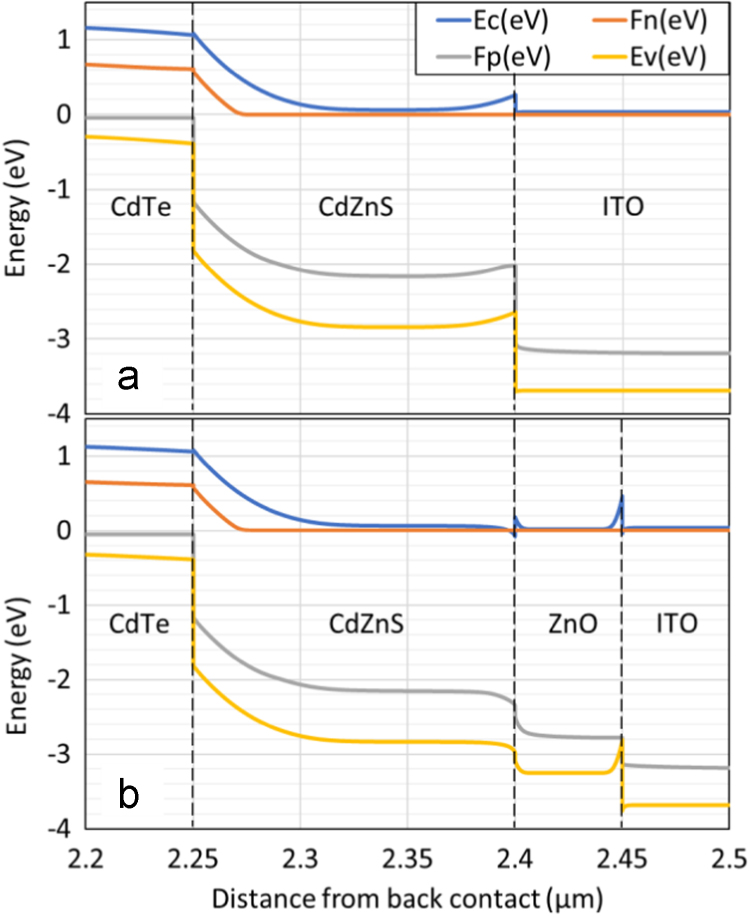
The band alignment of CdTe solar cells (a) without and (b) with a 50 nm ZnO layer sandwiched between the window (CdZnS) and front contact (ITO) layers. Spikes are introduced in the conduction band (*E*_c_) at the CdZnS/ZnO and ZnO/ITO interfaces.

Device simulations by SCAPS was carried out to inspect the band-alignment and calculate device parameters. The band-alignment of device structures with and without a 50 nm ZnO buffer film is given in Fig. 2. Small energy spikes are seen to be introduced in the conduction band at the layer interfaces to the ZnO film.

In device simulations, if the *N*_A_ is kept constant at 1 × 10^16^ cm^−3^, addition of the ZnO layer and associated the spikes in the conduction band near the buffer layer did not change the device *V*_oc_. When experimental *N*_A_ values from Fig. 1b (i.e. 1.0 × 10^16^ and 1.7 × 10^16^ cm^-3^ for the HRT and no HRT cases, respectively) are used instead, then a *V*_oc_ loss of ~22 mV was calculated for the cell with the HRT layer.

## 2 Experimental Design, Materials, And Methods

Arsenic profiling in the solar cells was collected with a Cameca IMS-4f secondary ion-mass spectrometer at LSA Ltd. Cs+ ions, at 10 keV energy obtained with 20 nA current, were used as the primary ions. The specimen was 1 × 1 cm^2^ in size, cleaved from the main coupon, and etched in 0.2% Br_2_ (in methanol) for 5 s to polish the sample surface and increase the depth resolution. A previously characterized CdTe:As layer (supplied by CSER to LSA Ltd.) was used for calibration.

CV data were collected from the solar cell structures in dark and at room temperature conditions using a Solartron Impedance Analyzer. An ac bias amplitude of 10 mV was applied at 300 kHz whilst the DC bias (*V*_appl_) was swept from -3V to +1 V. Linear part of the 1/*C*^2^ vs. *V* plot (near *V*_appl_ = 0 V) was then analyzed using the procedure described in Ref. [2], to extract the net acceptor density *N*_A_.

The SCAPS programme, used to simulate solar cell characteristics, is a one-dimensional solar cell simulation programme and freely available online through its owner, Prof. Marc Burgelman, University of Gent, Belgium [3]. The layer parameters used in these simulations are given in Refs. [1], [4].
